# Crystal structure of (*S*)-5-(3-acetyl-5-chloro-2-ethoxy-6-fluorophenyl)-2-oxazolidinone

**DOI:** 10.1107/S2056989024001920

**Published:** 2024-03-19

**Authors:** Victor Li, Glenn P. A. Yap, Chaoying Ni

**Affiliations:** a W. M. Keck Center for Advanced Microscopy and Microanalysis, University of Delaware, Newark, DE 19716, USA; b Department of Chemistry and Biochemistry, University of Delaware, Newark, DE 19716, USA; c Department of Materials Science and Engineering, University of Delaware, Newark, DE 19716, USA; Texas A & M University, USA

**Keywords:** crystal structure, oxazolidinone, parsaclib, pharmaceutical, kinase inhibitor, anti-cancer, drug

## Abstract

The structure of (*S*)-5-(3-acetyl-5-chloro-2-ethoxy-6-fluorophenyl)-2-oxazolidinone, C_13_H_13_ClFNO_4_, at 100 K has monoclinic (*P*2_1_) symmetry. The compound has a polymeric structure propagated by a screw axis parallel to the *b* axis with N—H⋯O hydrogen bonding. It is of inter­est with respect to efforts in the synthesis of a candidate anti­cancer drug, parsaclisib.

## Abstract 2

The structure of (*S*)-5-(3-acetyl-5-chloro-2-ethoxy-6-fluorophenyl)-2-oxazolidinone has been determined to establish its absolute configuration in efforts to synthesize an anti­cancer drug candidate, parsaclisib.

## 1. Chemical context

Oxazolidinones are a class of compounds containing the five-membered heterocycle 1,3-oxazolidin-2-one and were mainly used for anti­microbials acting as protein synthesis inhibitors targeting *N*-formyl­methionyl-tRNA to ribosome binding (Zhao *et al.*, 2021 ▸). Cases with elevated levels of phospho­inositide 3-kinase delta (PI3Kδ) were found associated with increased cancer susceptibility (Crank *et al.*, 2014 ▸). An oxa­zol­idinone drug candidate, (4*R*)-4-[3-[(1*S*)-1-(4-amino-3-methyl­pyrazolo­[3,4-*d*]pyrimidin-1-yl)eth­yl]-5-chloro-2-eth­oxy-6-fluoro­phen­yl]pyrrolidin-2-one, parsaclisib, was discovered to be a potent PI3Kδ inhibitor (Zinzani *et al.*, 2023 ▸). As part of evolving attempts to improve the synthesis of parsaclisib, we were sent samples of an inter­mediate product that required confirmation of substituents and absolute chirality determination. Our diffraction studies identified it as (*S*)-5-(3-acetyl-5-chloro-2-ethoxy-6-fluorophenyl)-2-oxazolidinone. Atom C-3 has been determined by our study to have *S* absolute chirality, which can yield the corresponding alcohol *via* enantioselective ketone reduction (Mao *et al.*, 2005 ▸), which should subsequently yield parsaclisib as per the reaction scheme. 
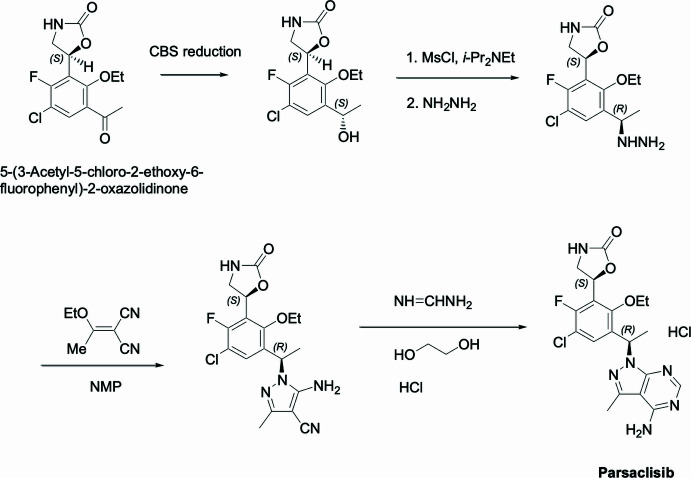


## 2. Structural commentary

**Figure 1:**
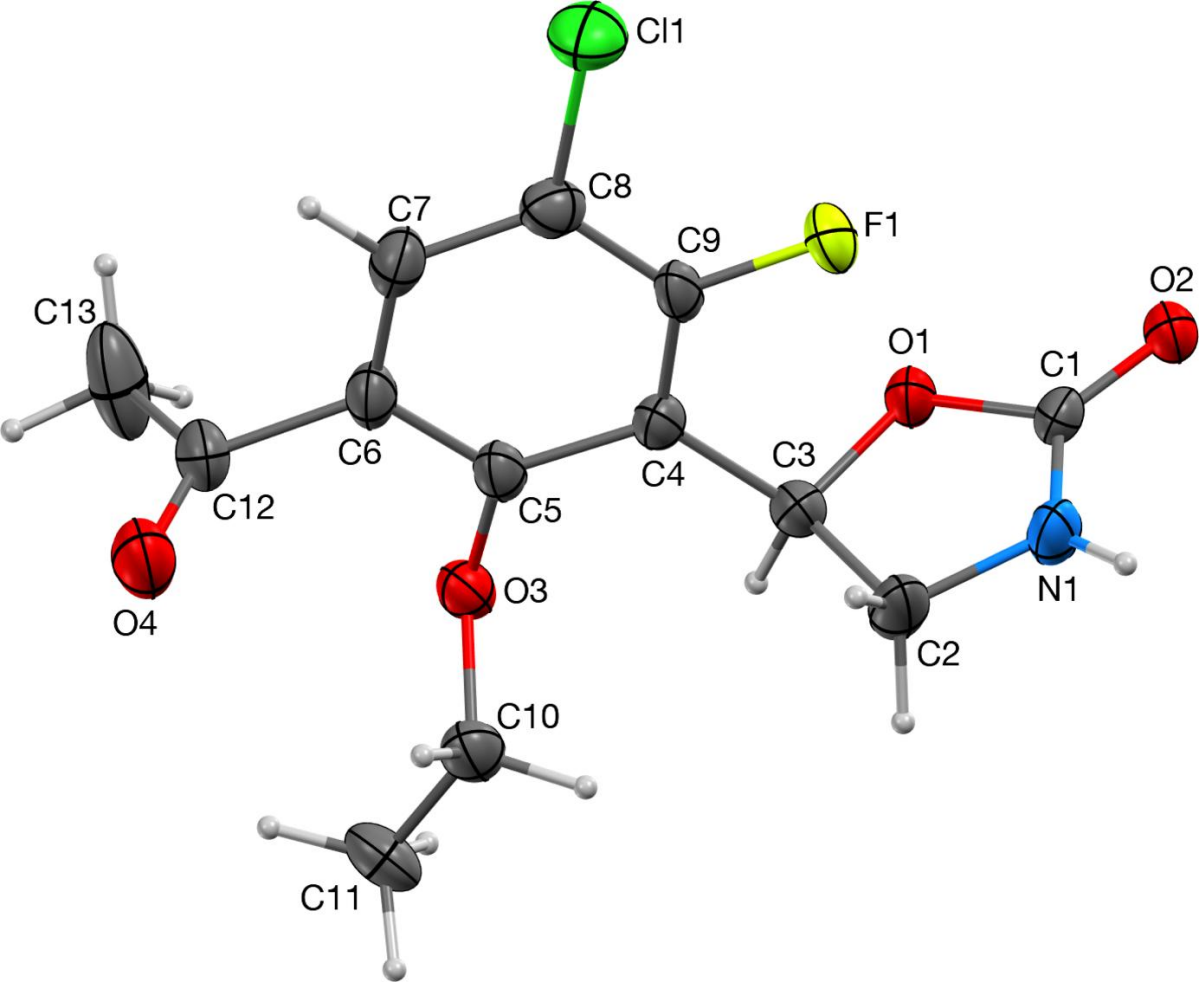
The contents of the asymmetric unit with atom labeling. H-atom labels are omitted for clarity. Displacement ellipsoids are plotted at 50% probability.

The asymmetric unit consisting of one complete mol­ecule of the title compound is shown in Fig. 1 ▸. Consistent with similar structures (*vide infra*), the oxazolidinone ring is essentially planar and is twisted from the plane of the dihalophenyl ring as seen from twist angles C2—C3—C4—C9 = 70.5 (5)°. One of the two symmetry-unique mol­ecules in (*R*)-5-mesityloxazolidin-2-one has the closest similar twist of 73.5 (2)° (Qin *et al.*, 2012 ▸). The acetate and eth­oxy groups in the title compound are almost perpendicular to the phenyl ring with torsion angles C7—C6—C12—O4 = −92.8 (6)° and C10—O3—C5—C6 = −96.8 (4)°, respectively. The absolute structure refined to nil indicating the correct handedness has been established.

## 3. Supra­molecular features

**Figure 2:**
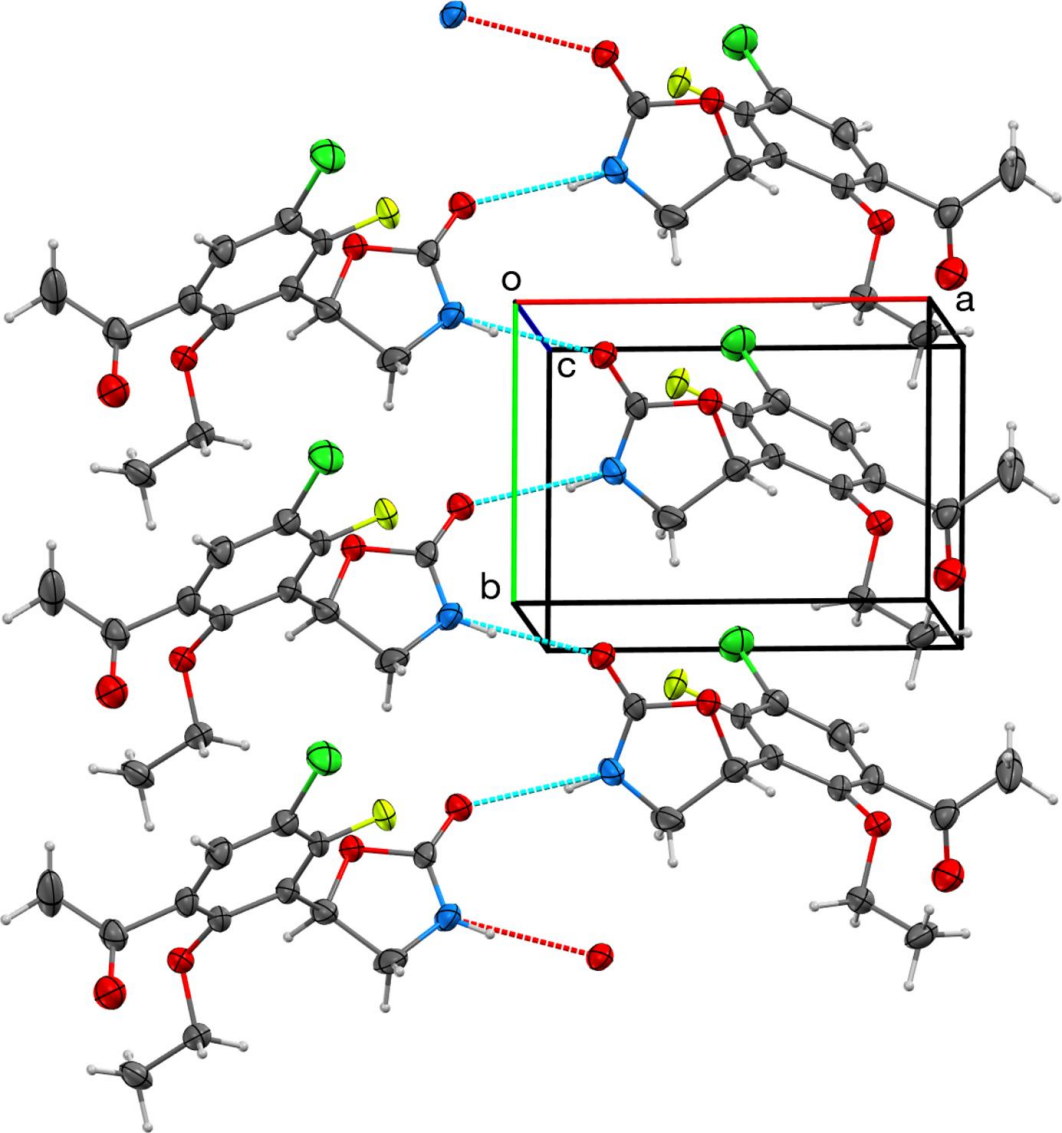
A view perpendicular to the *c* axis showing hydrogen-bonding inter­actions (dotted lines) forming a chain parallel to the *b* axis. Displacement ellipsoids are plotted at 50% probability.

In the crystal, N—H⋯O hydrogen-bonding inter­actions (Table 1 ▸) occur between neighboring mol­ecules related by −*x*, 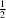 + *y*, 1 − *z*, resulting in chains parallel to the *b*-axis direction (Fig. 2 ▸). In contrast, di­chloro-{2-meth­oxy-4-[2-(pyridin-2-yl)-1,3-oxazolidin-5-yl]phenol}palladium aceto­nitrile solvate does not show this type of hydrogen bonding, perhaps because the oxazolidinone N atom is also coordinated to palladium (Denisov & Gagarskikh, 2021 ▸). Remarkably, the four other structures do display N—H⋯O hydrogen bonding; however, in each case, this leads to pair-wise dimer formation instead of a more extended structure (Chen *et al.*, 2021 ▸; Norte *et al.*, 1988 ▸; Qin *et al.*, 2012 ▸; Bresciani *et al.*, 2020 ▸).

## 4. Database survey

A search of the Cambridge Structural Database with WebCSD (https://www.ccdc.cam.ac.uk/structures/WebCSD, accessed November 8, 2023; Groom *et al.*, 2016 ▸) for structures containing the 5-(arene)-oxazolidine-2-one moiety yielded five additional structures: EWIPEI (Chen *et al.*, 2021 ▸), GIGHUZ (Norte *et al.*, 1988 ▸), MAZDEN (Qin *et al.*, 2012 ▸), WAFCEP (Bresciani *et al.*, 2020 ▸) and YALYUJ (Denisov & Gagarskikh, 2021 ▸).

## 5. Synthesis and crystallization

(*S*)-5-(3-Acetyl-5-chloro-2-ethoxy-6-fluorophenyl)-2-oxazol­id­inone and solvents were used as received without further purification. (*S*)-5-(3-Acetyl-5-chloro-2-eth­oxy-6-fluoro­phen­yl)-2-oxazolidinone (20 mg) was dissolved in a mixed solvent of methanol (3 mL) and di­chloro­methane (1 mL). The solution was allowed to evaporate slowly at room temperature until suitable crystals were deposited.

## 6. Refinement

Crystal data, data collection and structure refinement details are summarized in Table 2 ▸. Amide H atoms were located from difference maps and positionally refined. Other H atoms were positioned geometrically. All H atoms refined as riding with *U*_iso_(H) = 1.2 or 1.5*U*_eq_(C,N).

## Supplementary Material

Crystal structure: contains datablock(s) I. DOI: 10.1107/S2056989024001920/jy2039sup1.cif

Structure factors: contains datablock(s) I. DOI: 10.1107/S2056989024001920/jy2039Isup2.hkl

Supporting information file. DOI: 10.1107/S2056989024001920/jy2039Isup3.cml

CCDC reference: 2306123

Additional supporting information:  crystallographic information; 3D view; checkCIF report

## Figures and Tables

**Table 1:** Hydrogen-bond geometry (Å, °)

*D*—H⋯*A*	*D*—H	H⋯*A*	*D*⋯*A*	*D*—H⋯*A*
N1—H1⋯O2^i^	0.84 (6)	2.12 (6)	2.923 (4)	161 (5)

Symmetry code: (i) 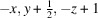 .

**Table 2:** Experimental details

Crystal data	
Chemical formula	C_13_H_13_ClFNO_4_
*M*_r_	301.69
Crystal system, space group	Monoclinic, *P*2_1_
Temperature (K)	100
*a*, *b*, *c* (Å)	7.8729 (12), 5.5655 (8), 15.492 (2)
β (°)	101.446 (3)
*V* (Å^3^)	665.31 (17)
*Z*	2
Radiation type	Cu *K*α
μ (mm^−1^)	2.80
Crystal size (mm)	0.41 × 0.10 × 0.08

Data collection	
Diffractometer	Bruker Venture Photon III
Absorption correction	Multi-scan (*SADABS*; Krause et al., 2015 ▸)
*T*_min_, *T*_max_	0.523, 0.754
No. of measured, independent and observed [*I* > 2σ(*I*)] reflections	12247, 2540, 2483
*R*_int_	0.061
(sin θ/λ)_max_ (Å^−1^)	0.620

Refinement	
*R*[*F* ^2^ > 2σ(*F* ^2^)], *wR*(*F* ^2^), *S*	0.050, 0.136, 1.08
No. of reflections	2540
No. of parameters	186
No. of restraints	1
H-atom treatment	H atoms treated by a mixture of independent and constrained refinement
Δρ_max_, Δρ_min_ (e Å^−3^)	0.44, −0.28
Absolute structure	Flack *x* determined using 1052 quotients [(*I* ^+^)−(*I* ^−^)]/[(*I* ^+^)+(*I* ^−^)] (Parsons *et al.*, 2013 ▸)
Absolute structure parameter	0.009 (11)

Computer programs: *APEX4* (Bruker, 2021 ▸), *SAINT* (Bruker, 2016 ▸), *SHELXT2018/2* (Sheldrick, 2015*a* ▸), *SHELXL2019/3* (Sheldrick, 2015*b* ▸), and *OLEX2* (Dolomanov *et al.*, 2009 ▸).

## supplementary crystallographic information

### Crystal data

**Table:**

C_13_H_13_ClFNO_4_	*F*(000) = 312
*M**_r_* = 301.69	*D*_x_ = 1.506 Mg m^−^^3^
Monoclinic, *P*2_1_	Cu *K*α radiation, λ = 1.54178 Å
*a* = 7.8729 (12) Å	Cell parameters from 9877 reflections
*b* = 5.5655 (8) Å	θ = 2.9–72.0°
*c* = 15.492 (2) Å	µ = 2.80 mm^−^^1^
β = 101.446 (3)°	*T* = 100 K
*V* = 665.31 (17) Å^3^	Needle, colourless
*Z* = 2	0.41 × 0.10 × 0.08 mm

### Data collection

**Table:**

Bruker Venture Photon III diffractometer	2483 reflections with *I* > 2σ(*I*)
area detector profiles from φ and ω scans	*R*_int_ = 0.061
Absorption correction: multi-scan (SADABS; Krause et al., 2015)	θ_max_ = 72.9°, θ_min_ = 2.9°
*T*_min_ = 0.523, *T*_max_ = 0.754	*h* = −9→9
12247 measured reflections	*k* = −6→6
2540 independent reflections	*l* = −19→19

### Refinement

**Table:**

Refinement on *F*^2^	Hydrogen site location: mixed
Least-squares matrix: full	H atoms treated by a mixture of independent and constrained refinement
*R*[*F*^2^ > 2σ(*F*^2^)] = 0.050	*w* = 1/[σ^2^(*F*_o_^2^) + (0.0799*P*)^2^ + 0.4134*P*] where *P* = (*F*_o_^2^ + 2*F*_c_^2^)/3
*wR*(*F*^2^) = 0.136	(Δ/σ)_max_ < 0.001
*S* = 1.08	Δρ_max_ = 0.44 e Å^−^^3^
2540 reflections	Δρ_min_ = −0.28 e Å^−^^3^
186 parameters	Absolute structure: Flack *x* determined using 1052 quotients [(*I*^+^)-(*I*^-^)]/[(*I*^+^)+(*I*^-^)] (Parsons *et al.*, 2013)
1 restraint	Absolute structure parameter: 0.009 (11)
Primary atom site location: dual	

### Special details

**Table:**

Geometry. All esds (except the esd in the dihedral angle between two l.s. planes) are estimated using the full covariance matrix. The cell esds are taken into account individually in the estimation of esds in distances, angles and torsion angles; correlations between esds in cell parameters are only used when they are defined by crystal symmetry. An approximate (isotropic) treatment of cell esds is used for estimating esds involving l.s. planes.
Refinement. 1. Fixed Uiso At 1.2 times of: All C(H) groups, All C(H,H) groups, All N(H) groups At 1.5 times of: All C(H,H,H) groups 2.a Ternary CH refined with riding coordinates: C3(H3) 2.b Secondary CH2 refined with riding coordinates: C2(H2A,H2B), C10(H10A,H10B) 2.c Aromatic/amide H refined with riding coordinates: C7(H7) 2.d Idealised Me refined as rotating group: C11(H11A,H11B,H11C), C13(H13A,H13B,H13C)

### Fractional atomic coordinates and isotropic or equivalent isotropic displacement parameters (Å^2^)

**Table:**

	*x*	*y*	*z*	*U*_iso_*/*U*_eq_	
Cl1	0.46325 (14)	−0.0030 (2)	0.91489 (7)	0.0449 (3)	
F1	0.3318 (3)	0.1581 (4)	0.73539 (17)	0.0349 (6)	
O1	0.4236 (3)	0.2441 (5)	0.57659 (19)	0.0301 (6)	
O2	0.1716 (4)	0.0984 (5)	0.50190 (19)	0.0322 (7)	
O3	0.8242 (3)	0.6319 (5)	0.70925 (19)	0.0289 (6)	
O4	0.9772 (4)	0.7707 (7)	0.9207 (2)	0.0495 (9)	
N1	0.1913 (5)	0.4736 (7)	0.5652 (2)	0.0353 (8)	
H1	0.090 (7)	0.528 (11)	0.557 (3)	0.042*	
C1	0.2492 (5)	0.2622 (7)	0.5438 (3)	0.0268 (8)	
C2	0.3247 (6)	0.6254 (8)	0.6146 (4)	0.0493 (13)	
H2A	0.298858	0.669061	0.672563	0.059*	
H2B	0.339997	0.773721	0.581675	0.059*	
C3	0.4851 (5)	0.4609 (7)	0.6246 (3)	0.0328 (9)	
H3	0.573730	0.539348	0.595815	0.039*	
C4	0.5655 (5)	0.4054 (7)	0.7189 (3)	0.0270 (8)	
C5	0.7273 (5)	0.5032 (8)	0.7582 (3)	0.0275 (8)	
C6	0.7992 (5)	0.4604 (8)	0.8463 (3)	0.0294 (8)	
C7	0.7180 (6)	0.3066 (8)	0.8952 (3)	0.0321 (9)	
H7	0.769301	0.272687	0.954743	0.039*	
C8	0.5617 (5)	0.2021 (8)	0.8570 (3)	0.0304 (9)	
C9	0.4874 (5)	0.2588 (7)	0.7713 (3)	0.0278 (8)	
C10	0.7841 (5)	0.8849 (8)	0.7014 (3)	0.0353 (10)	
H10A	0.766791	0.949590	0.758510	0.042*	
H10B	0.676577	0.911158	0.656942	0.042*	
C11	0.9338 (6)	1.0079 (9)	0.6736 (3)	0.0387 (10)	
H11A	0.957185	0.931322	0.620211	0.058*	
H11B	1.036659	0.995249	0.720835	0.058*	
H11C	0.905624	1.177735	0.661648	0.058*	
C12	0.9720 (6)	0.5739 (8)	0.8859 (3)	0.0345 (9)	
C13	1.1270 (6)	0.4320 (13)	0.8800 (4)	0.0605 (18)	
H13A	1.231157	0.523590	0.905643	0.091*	
H13B	1.127680	0.397057	0.818083	0.091*	
H13C	1.125416	0.281004	0.912361	0.091*	

### Atomic displacement parameters (Å^2^)

**Table:**

	*U*^11^	*U*^22^	*U*^33^	*U*^12^	*U*^13^	*U*^23^
Cl1	0.0441 (6)	0.0441 (6)	0.0467 (6)	−0.0063 (5)	0.0097 (5)	0.0123 (5)
F1	0.0204 (11)	0.0352 (14)	0.0458 (14)	−0.0082 (9)	−0.0014 (9)	0.0003 (11)
O1	0.0236 (13)	0.0249 (14)	0.0366 (15)	−0.0006 (11)	−0.0063 (11)	−0.0011 (11)
O2	0.0284 (14)	0.0259 (14)	0.0369 (15)	−0.0028 (11)	−0.0062 (12)	−0.0021 (12)
O3	0.0246 (13)	0.0234 (14)	0.0374 (15)	−0.0009 (11)	0.0035 (11)	−0.0027 (12)
O4	0.0407 (18)	0.040 (2)	0.059 (2)	−0.0019 (15)	−0.0109 (16)	−0.0079 (16)
N1	0.0272 (17)	0.0273 (18)	0.044 (2)	0.0064 (16)	−0.0101 (14)	−0.0030 (16)
C1	0.0214 (18)	0.0269 (19)	0.0282 (19)	0.0005 (15)	−0.0043 (14)	0.0061 (15)
C2	0.047 (3)	0.023 (2)	0.063 (3)	0.007 (2)	−0.024 (2)	−0.003 (2)
C3	0.031 (2)	0.023 (2)	0.039 (2)	−0.0033 (16)	−0.0072 (17)	0.0025 (16)
C4	0.0220 (17)	0.0228 (17)	0.033 (2)	0.0010 (14)	−0.0024 (15)	0.0008 (15)
C5	0.0199 (16)	0.0234 (17)	0.036 (2)	0.0001 (15)	−0.0007 (14)	−0.0027 (17)
C6	0.0216 (17)	0.031 (2)	0.0322 (19)	0.0010 (15)	−0.0037 (14)	−0.0037 (16)
C7	0.0283 (19)	0.037 (2)	0.0278 (19)	0.0033 (16)	−0.0029 (16)	−0.0010 (16)
C8	0.0275 (19)	0.027 (2)	0.037 (2)	0.0023 (15)	0.0073 (16)	0.0039 (16)
C9	0.0158 (16)	0.0233 (18)	0.041 (2)	−0.0003 (14)	−0.0035 (15)	−0.0026 (16)
C10	0.029 (2)	0.025 (2)	0.049 (3)	0.0024 (17)	0.0004 (18)	−0.0005 (19)
C11	0.041 (2)	0.028 (2)	0.048 (2)	−0.0125 (19)	0.0126 (19)	−0.008 (2)
C12	0.030 (2)	0.036 (2)	0.032 (2)	−0.0060 (17)	−0.0048 (16)	−0.0022 (17)
C13	0.025 (2)	0.077 (4)	0.075 (4)	−0.002 (2)	0.001 (2)	−0.039 (3)

### Geometric parameters (Å, º)

**Table:**

Cl1—C8	1.727 (4)	C4—C9	1.379 (6)
F1—C9	1.361 (4)	C5—C6	1.392 (6)
O1—C1	1.370 (5)	C6—C7	1.380 (6)
O1—C3	1.449 (5)	C6—C12	1.515 (5)
O2—C1	1.211 (5)	C7—H7	0.9500
O3—C5	1.378 (5)	C7—C8	1.383 (6)
O3—C10	1.443 (5)	C8—C9	1.377 (6)
O4—C12	1.217 (6)	C10—H10A	0.9900
N1—H1	0.84 (6)	C10—H10B	0.9900
N1—C1	1.328 (6)	C10—C11	1.498 (6)
N1—C2	1.443 (6)	C11—H11A	0.9800
C2—H2A	0.9900	C11—H11B	0.9800
C2—H2B	0.9900	C11—H11C	0.9800
C2—C3	1.542 (6)	C12—C13	1.472 (7)
C3—H3	1.0000	C13—H13A	0.9800
C3—C4	1.505 (5)	C13—H13B	0.9800
C4—C5	1.408 (5)	C13—H13C	0.9800
			
C1—O1—C3	109.7 (3)	C6—C7—C8	119.7 (4)
C5—O3—C10	114.7 (3)	C8—C7—H7	120.1
C1—N1—H1	130 (4)	C7—C8—Cl1	120.6 (3)
C1—N1—C2	113.7 (4)	C9—C8—Cl1	120.2 (3)
C2—N1—H1	116 (4)	C9—C8—C7	119.2 (4)
O2—C1—O1	120.3 (4)	F1—C9—C4	118.2 (3)
O2—C1—N1	129.7 (4)	F1—C9—C8	118.4 (4)
N1—C1—O1	109.9 (3)	C8—C9—C4	123.3 (4)
N1—C2—H2A	111.4	O3—C10—H10A	110.2
N1—C2—H2B	111.4	O3—C10—H10B	110.2
N1—C2—C3	101.6 (4)	O3—C10—C11	107.4 (4)
H2A—C2—H2B	109.3	H10A—C10—H10B	108.5
C3—C2—H2A	111.4	C11—C10—H10A	110.2
C3—C2—H2B	111.4	C11—C10—H10B	110.2
O1—C3—C2	105.0 (3)	C10—C11—H11A	109.5
O1—C3—H3	109.0	C10—C11—H11B	109.5
O1—C3—C4	111.1 (3)	C10—C11—H11C	109.5
C2—C3—H3	109.0	H11A—C11—H11B	109.5
C4—C3—C2	113.4 (4)	H11A—C11—H11C	109.5
C4—C3—H3	109.0	H11B—C11—H11C	109.5
C5—C4—C3	120.7 (4)	O4—C12—C6	120.2 (4)
C9—C4—C3	122.9 (3)	O4—C12—C13	123.7 (4)
C9—C4—C5	116.5 (3)	C13—C12—C6	116.1 (4)
O3—C5—C4	121.1 (3)	C12—C13—H13A	109.5
O3—C5—C6	117.8 (3)	C12—C13—H13B	109.5
C6—C5—C4	121.0 (4)	C12—C13—H13C	109.5
C5—C6—C12	118.9 (4)	H13A—C13—H13B	109.5
C7—C6—C5	120.1 (4)	H13A—C13—H13C	109.5
C7—C6—C12	120.9 (4)	H13B—C13—H13C	109.5
C6—C7—H7	120.1		
			
Cl1—C8—C9—F1	3.0 (5)	C3—C4—C9—C8	178.4 (4)
Cl1—C8—C9—C4	−174.5 (3)	C4—C5—C6—C7	4.6 (6)
O1—C3—C4—C5	132.4 (4)	C4—C5—C6—C12	−178.7 (4)
O1—C3—C4—C9	−47.5 (5)	C5—O3—C10—C11	161.8 (3)
O3—C5—C6—C7	−171.5 (4)	C5—C4—C9—F1	−179.1 (3)
O3—C5—C6—C12	5.1 (6)	C5—C4—C9—C8	−1.6 (6)
N1—C2—C3—O1	2.5 (5)	C5—C6—C7—C8	−2.3 (6)
N1—C2—C3—C4	−119.0 (4)	C5—C6—C12—O4	90.6 (5)
C1—O1—C3—C2	−2.1 (5)	C5—C6—C12—C13	−89.9 (6)
C1—O1—C3—C4	120.9 (4)	C6—C7—C8—Cl1	176.6 (3)
C1—N1—C2—C3	−2.3 (6)	C6—C7—C8—C9	−1.7 (6)
C2—N1—C1—O1	1.1 (5)	C7—C6—C12—O4	−92.8 (6)
C2—N1—C1—O2	−178.7 (5)	C7—C6—C12—C13	86.7 (6)
C2—C3—C4—C5	−109.5 (4)	C7—C8—C9—F1	−178.7 (4)
C2—C3—C4—C9	70.5 (5)	C7—C8—C9—C4	3.8 (6)
C3—O1—C1—O2	−179.4 (4)	C9—C4—C5—O3	173.4 (4)
C3—O1—C1—N1	0.8 (4)	C9—C4—C5—C6	−2.6 (6)
C3—C4—C5—O3	−6.6 (6)	C10—O3—C5—C4	87.1 (5)
C3—C4—C5—C6	177.4 (4)	C10—O3—C5—C6	−96.8 (4)
C3—C4—C9—F1	0.9 (6)	C12—C6—C7—C8	−178.9 (4)

### Hydrogen-bond geometry (Å, º)

**Table:**

*D*—H···*A*	*D*—H	H···*A*	*D*···*A*	*D*—H···*A*
N1—H1···O2^i^	0.84 (6)	2.12 (6)	2.923 (4)	161 (5)

Symmetry code: (i) −*x*, *y*+1/2, −*z*+1.
